# A nearly on-axis spectroscopic system for simultaneously measuring UV–visible absorption and X-ray diffraction in the SPring-8 structural genomics beamline

**DOI:** 10.1107/S1600577515018275

**Published:** 2016-01-01

**Authors:** Miyuki Sakaguchi, Tetsunari Kimura, Takuma Nishida, Takehiko Tosha, Hiroshi Sugimoto, Yoshihiro Yamaguchi, Sachiko Yanagisawa, Go Ueno, Hironori Murakami, Hideo Ago, Masaki Yamamoto, Takashi Ogura, Yoshitsugu Shiro, Minoru Kubo

**Affiliations:** a Graduate School of Life Science, University of Hyogo, 3-2-1 Kouto, Kamigori, Akoh, Hyogo 678-1297, Japan; b RIKEN SPring-8 Center, 1-1-1 Kouto, Sayo, Hyogo 679-5148, Japan; c PRESTO, Japan Science and Technology Agency, 4-1-8 Honcho, Kawaguchi, Saitama 332-0012, Japan

**Keywords:** cytochrome P450nor, UV–visible absorption spectroscopy, radiation damage, simultaneous measurement, X-ray crystallography

## Abstract

UV–visible absorption spectroscopy is useful for probing the electronic and structural changes of protein active sites, and thus the on-line combination of X-ray diffraction and spectroscopic analysis is increasingly being applied. Herein, a novel absorption spectrometer was developed at SPring-8 BL26B2 with a nearly on-axis geometry between the X-ray and optical axes. A small prism mirror was placed near the X-ray beamstop to pass the light only 2° off the X-ray beam, enabling spectroscopic analysis of the X-ray-exposed volume of a crystal during X-ray diffraction data collection. The spectrometer was applied to NO reductase, a heme enzyme that catalyzes NO reduction to N_2_O. Radiation damage to the heme was monitored in real time during X-ray irradiation by evaluating the absorption spectral changes. Moreover, NO binding to the heme was probed *via* caged NO photolysis with UV light, demonstrating the extended capability of the spectrometer for intermediate analysis.

## Abstract 2

A nearly on-axis UV–visible absorption spectrometer was developed at SPring-8 that enables spectroscopic analysis of the X-ray-exposed volume of a crystal during X-ray diffraction data collection.

## 1. Introduction   

X-ray crystallography is one of the most powerful tools for protein structural analysis at the atomic level and has been extensively used in structural biology. However, X-ray radiation damage has been a problem for accurate structural determination, particularly for metalloproteins, because the metal centers are sensitive to hydrated electrons (Garman & Owen, 2006 ▸; Yano *et al.*, 2005 ▸). Because spectroscopy is useful for determining the physicochemical properties of proteins, spectroscopic analysis of a crystalline sample has been complementarily combined with X-ray crystallography to evaluate the sample status. Although Raman and fluorescence spectroscopies have been recently used for this purpose (Bourgeois *et al.*, 2002 ▸; Carpentier *et al.*, 2007 ▸; Davies *et al.*, 2009 ▸; Klink *et al.*, 2006 ▸; Orville *et al.*, 2011 ▸; Pompidor *et al.*, 2013 ▸; Royant *et al.*, 2007 ▸; Stoner-Ma *et al.*, 2011 ▸), UV–visible absorption spectroscopy is more readily available and effective for probing the redox states of metal centers. Moreover, it can be used to identify intermediate species during the study of protein dynamics (De la Mora-Rey & Wilmot, 2007 ▸).

UV–visible absorption spectrometers have previously been installed on-line at synchrotron facilities, including APS (Pearson *et al.*, 2007 ▸), ESRF (McGeehan *et al.*, 2009 ▸; Royant *et al.*, 2007 ▸), NSLS (Orville *et al.*, 2011 ▸), SLS (Beitlich *et al.*, 2007 ▸), SPring-8 (Sakai *et al.*, 2002 ▸; Aoyama *et al.*, 2009 ▸) and SSRL (Meharenna *et al.*, 2010 ▸). In these spectrometers, UV–visible white light is introduced off-axis from the X-ray beam. The off-axis geometry prevents the spectroscopic analysis from interfering with the X-ray diffraction data collection. In this geometry, however, it is often difficult to monitor the X-ray-exposed volume of a crystal when the crystal is larger than the X-ray beam size. To solve this problem, an on-axis geometry was recently adopted at SLS (Owen *et al.*, 2009 ▸; Pompidor *et al.*, 2013 ▸; Fuchs *et al.*, 2014 ▸) and SPring-8 (Shimizu *et al.*, 2013 ▸). However, these on-axis systems suffer from a fundamental drawback: the optical setup blocks the diffracted X-rays and disturbs the X-ray diffraction analyses.

Herein, a novel absorption spectrometer that adopts a nearly on-axis geometry but which does not interfere with X-ray diffraction data collection when operating is described. In this spectrometer, a small prism mirror was placed near the X-ray beamstop that passes the white light to a crystal nearly collinear to (only 2° off) the X-ray beam and minimizes the interference with the X-rays diffracted from the crystal. To evaluate the performance of this system, the spectrometer was installed at the structural genomics beamline BL26B2 at SPring-8 and used to monitor in real time the radiation damage of fungal NO reductase (P450nor), a heme enzyme responsible for reductive elimination of NO in the denitrification process (Shoun *et al.*, 2012 ▸). In addition, the spectrometer was used to monitor NO binding to the heme of P450nor upon caged NO photolysis in the crystalline state, demonstrating the potential of the spectrometer for intermediate analysis.

## 2. Instrumentation   

The absorption spectrometer consists of a white light source, an optical system and a detector. The white light can be supplied by a high-power white LED (UHP-Mic-LED-White, Pritzmatix) or an Xe flash lamp (L11316-11-11, Hamamatsu Photonics). The white LED is a simple CW light source at visible wavelengths (420–700 nm) and was used here for the visible absorption spectroscopy of P450nor. On the other hand, the Xe-flash lamp emits microsecond pulses covering UV to near-IR ranges, though the available wavelength is limited to between 390 and 850 nm owing to the optics specifications used in the current setup. Both of the light sources provide the fiber-coupled output [200 µm-diameter core, 0.22 numerical aperture]. After collimation, the white light is focused into a 25 µm-diameter pinhole and collimated again for spatial filtering, which helps to decrease the focal size of the white light at the sample point. An optical beam shutter (SH05, Thorlabs) is placed in the white light path and synchronized with the X-ray beam shutter.

**Figure 1:**
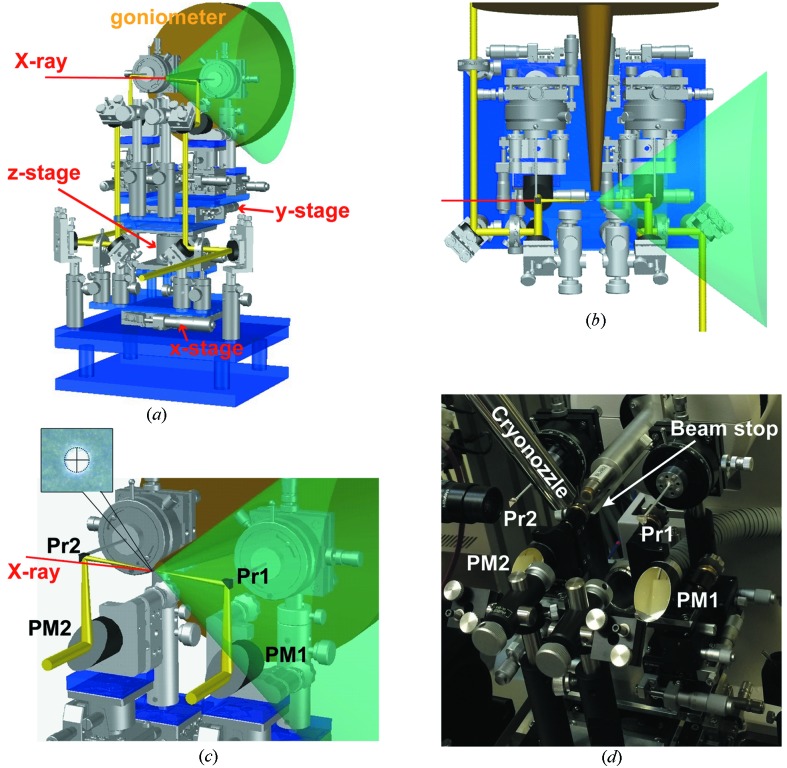
Nearly on-axis absorption spectrometer. (*a*) Three-dimensional drawing of the optical system with a goniometer (dark yellow). The transparent green cone represents the path of the diffracted X-rays (corresponding to 1.9 Å resolution, collected at 10 keV). The white light path is shown in yellow. The white light focal point is adjusted to a crystal sample using the *x*, *y* and *z* stages. The white light beam path is 2° off the X-ray beam (red line). The X-ray beamstop is omitted from the drawing. (*b*) Top view of the optical system. (*c*) Enlarged view around the sample point. Pr1 and Pr2: prism mirrors; PM1 and PM2: 90° off-axis parabolic mirrors. The circular focal spot image (100 µm diameter) of white light on a polyester film is shown in the inset. (*d*) Photograph of the optical system, including the cryo-nozzle and the X-ray beamstop.

The collimated white light (yellow beam path) is introduced into the optical system coupled to an X-ray diffractometer, as depicted in Figs. 1 ▸(*a*) and 1(*b*). The diffracted X-rays from a protein crystal loop-mounted on a goniometer are depicted by the transparent green cone. An enlarged view near the crystal is illustrated in Fig. 1 ▸(*c*) and a photograph is shown in Fig. 1 ▸(*d*). The white light is focused using a 90° off-axis parabolic mirror PM1 (*f* 101.6 mm) and then directed to the sample by a small prism mirror Pr1 (5 mm × 5 mm), which is mounted on a θ stage. The distance between the prism mirror and the sample is approximately 50 mm. To avoid interference between the white light and X-ray beamstop near the sample, the prism mirror is slightly offset from the X-ray beam height and rotated. The angle between the white light and X-ray beam axes is 2°. The diameter of the white light beam at the sample point is 100 µm, as determined from the beam spot image on a polyester film mounted on the goniometer [Fig. 1 ▸(*c*), inset]. The transmitted light from the sample is collected using a small prism mirror Pr2 and a 90° off-axis parabolic mirror PM2 (Fig. 1 ▸ *c*).

Absorption spectra are obtained using a fiber-coupled spectrometer (USB2000+, Ocean Optics) equipped with an optical fiber (P400-2-UV/VIS, Ocean Optics), a 500 nm-blazed, 600 grooves mm^−1^ grating, a long-pass filter for cutting the second-order light, a 2048-element CCD (Sony ILX511B) and a 16-bit AD converter. Note that the available wavelength can be expanded to the UV region (∼280 nm) by replacement of the optical fiber and the spectrograph grating. The spectral measurements are managed using LabVIEW (LabVIEW2012, National Instruments) on a personal computer that is controlled out of the experimental hatch. The spectral data acquisition is triggered by a TTL signal generated when the X-ray beam shutter is opened by the beamline control software BSS (Ueno *et al.*, 2005 ▸). The measurable optical density (OD) is ≤2.

## 3. Sample preparation   

### 3.1. Crystallization of P450nor   

P450nor from fungus *Fusarium oxysporum* was expressed in *E. coli* strain BL21(DE3) and purified using a reported method (Shimizu *et al.*, 2000 ▸). The purified sample with a *A*_413_/*A*_280_ ratio greater than 1.8 was dissolved in 20 m*M* potassium phosphate buffer (pH 6.0) with 10% (*v*/*v*) glycerol and stored at 193 K until used. As previously reported, single crystals in the ferric state were grown using the sitting drop vapor diffusion method in drops containing 1 µL of 25–50 mg ml^−1^ P450nor solution mixed with an equal volume of the precipitant solution [90 m*M* MES buffer (pH 6.0), 28–36% (*w*/*v*) PEG 4000, and 10% (*v*/*v*) glycerol] at 293 K (Park *et al.*, 1997 ▸). Typically, thin plate-like crystals were obtained within a few days.

### 3.2. Preparation of NO-bound ferric P450nor   

NO-bound ferric crystals were prepared using NO gas by the established method (Shimizu *et al.*, 2000 ▸). Ferric crystals were soaked in a cryo-solution [90 m*M* MES buffer (pH 6.0), 36% (*w*/*v*) PEG 4000 and 10% (*v*/*v*) glycerol] under an NO atmosphere for 10 min and then flash frozen in liquid N_2_. NO-bound ferric crystals were also prepared on-line using caged NO [*N*,*N*′-bis-(carboxymethyl)-*N*,*N*′-dinitroso-*p*-phenylenediamine], which releases NO upon UV irradiation (Namiki *et al.*, 1997 ▸). The caged NO powder was purchased from Dojindo and used without further purification. Ferric crystals were soaked in the cryo-solution containing 50 m*M* caged NO for 10 min and then flash frozen in liquid N_2_. A frozen crystal was then transferred to the beamline, loop-mounted on a goniometer at 100 K under cryogenic N_2_ gas and irradiated with UV light (337.1 nm, 3 ns pulse, 30 µJ) from an N_2_ gas laser (MNL308, LTB Lasertechnik Berlin). Note that the UV light was introduced on the same path as the white light using a flip mirror and its beam size at the sample was adjusted to be comparable with the size of the crystal by placing a lens in front of the flip mirror. First, one side of the crystal was irradiated with 50000 UV pulses, and then the crystal was rotated by 180° and the other side also irradiated with 50000 pulses. Finally, the crystal was annealed by blocking the cryogenic N_2_ stream for 3 s.

## 4. Applications   

**Figure 2:**
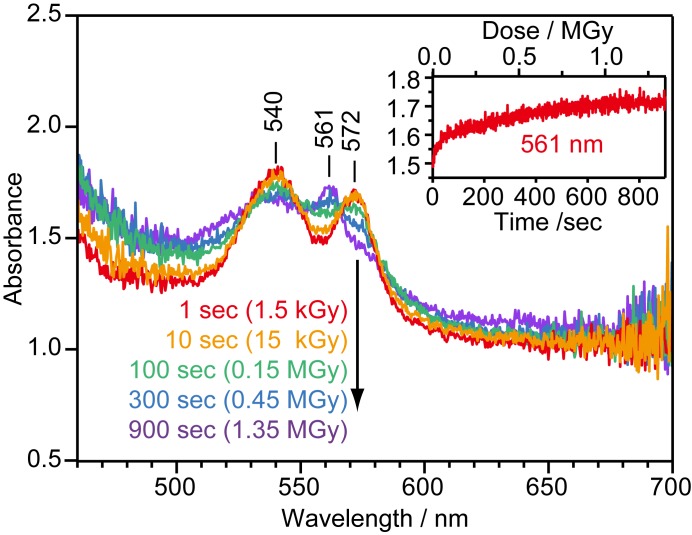
Absorption spectral changes during X-ray irradiation of the heme in an NO-bound ferric P450nor crystal at 100 K. The spectra with 60 ms exposure were obtained every 1 s after initiation of X-ray irradiation. Five spectra with X-ray irradiation times of 1 s (red), 10 s (orange), 100 s (green), 300 s (blue) and 900 s (purple) are shown. The temporal change in the absorbance at 561 nm is shown in the inset. The temporal change was fitted with a double exponential function with time constants of 16 and 400 s.

In the first application, the visible absorption spectrum of the heme in an NO-bound ferric P450nor crystal was monitored during X-ray irradiation (1.0 Å, 5.7 × 10^10^ photon flux) in real time at 100 K (Fig. 2 ▸). The X-ray dose was 1.5 kGy s^−1^, which was determined using *RADDOSE* (Paithankar *et al.*, 2009 ▸). The X-ray beam size was adjusted to 120 µm × 120 µm, which was comparable with the white light focal beam size (∅100 µm) at the crystal. In this study, the approximately 300 µm × 300 µm × 30 µm crystal was mounted such that the 30 µm thickness was the light path length, which is a suitable value for visible absorption spectroscopy given the 16 m*M* heme concentration in the crystal. The spectra exhibit an offset of ∼1 OD, probably because of light scattering on the crystal surface. A spectral change is observed during X-ray irradiation. Two absorption peaks at 540 nm and 572 nm, which are characteristic of the NO-bound ferric form (Fig. S1 of the supporting information), are broadened and diminished, respectively, and a new peak appears at 561 nm. Note that isosbestic points occur in the spectra, which implies that only two species (initial form and damaged form) are involved in the spectral change. The temporal absorbance change at 561 nm is plotted in the inset of Fig. 2 ▸, which successfully captures the evolution of radiation damage in real time.

**Figure 3:**
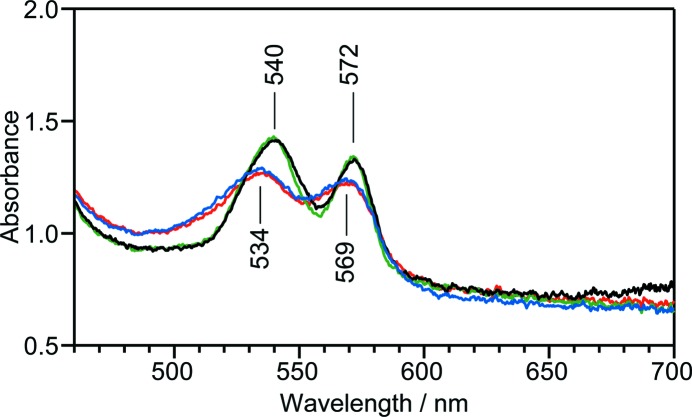
Visible absorption spectra of a ferric P450nor crystal containing caged NO at 100 K before UV irradiation (red), after UV irradiation (blue) and after annealing for 3 s (green). The spectrum of an NO-bound ferric P450nor crystal prepared with NO gas is shown in black as a reference. A linear background subtraction was performed to overlay the spectra for comparison. Each spectrum shown is the average of 500 data, each obtained with 60 ms exposure.

In the second application, light-induced NO binding to ferric P450nor was monitored using caged NO (Fig. 3 ▸). To trigger the photolysis of the caged NO, UV light was introduced to the optical path of the white light. The experimental details are described in §3. The absorption spectrum of a ferric P450nor crystal containing caged NO has two peaks at 534 and 569 nm, which is typical of the ferric state (Fig. 3 ▸, red). Note that caged NO has no absorption in the visible region (Namiki *et al.*, 1997 ▸). UV irradiation at 100 K does not change the absorption spectrum (Fig. 3 ▸, blue), because molecular diffusion is suppressed in the solvent space within the crystal at 100 K. Therefore, to allow the NO molecules to diffuse, the crystal is annealed up to room temperature for 3 s, which results in the spectral changes (Fig. 3 ▸, green) to the absorption spectrum of the NO-bound ferric form (Fig. 3 ▸, black). These observations demonstrate that our equipment is available for the photo-triggering of reactions and the subsequent sample monitoring during different reaction stages.

**Figure 4:**
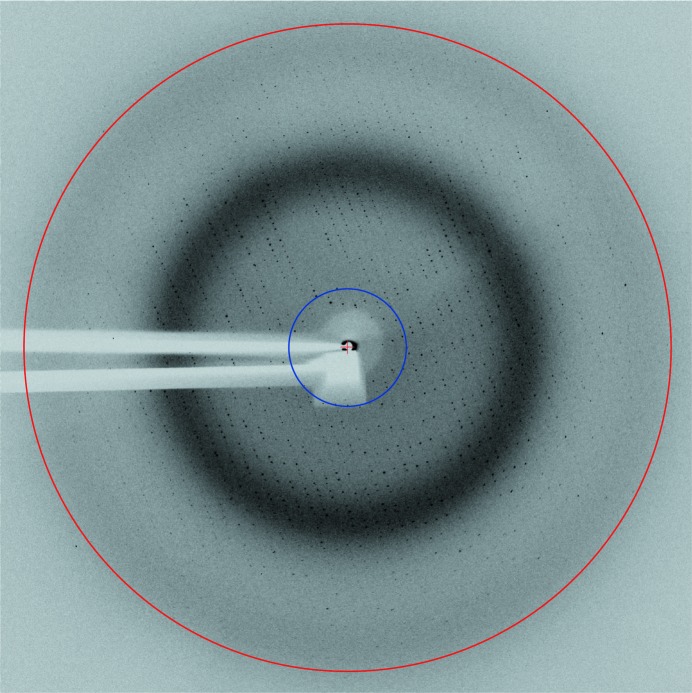
X-ray diffraction image of NO-bound P450nor in the presence of the nearly on-axis optical setup. The red and the blue circles represent resolutions of 2.0 and 10.0 Å, respectively. The image was taken with an X-ray exposure time of 4 s.

Lastly, an X-ray diffraction image of an NO-bound P450nor crystal was recorded to confirm the capability for X-ray diffraction data collection in the nearly on-axis optical geometry (Fig. 4 ▸). A square shadow at the center of the image is observed because of the small prism mirror (Fig. 1 ▸, Pr1) under the shadow of the X-ray beamstop. Nevertheless, a pattern of diffraction spots is successfully obtained, and the presence of the prism shadow has no significant effect on the completeness and the redundancy of the full data set (Table S1, Fig. S2), indicative of the advantage of the nearly on-axis geometry for simultaneous X-ray diffraction data collection and spectroscopic analysis.

## 5. Conclusions   

An on-line absorption spectrometer with a nearly on-axis geometry for the X-ray and optical axes has been designed and installed at SPring-8 BL26B2 that allows the collection of X-ray diffraction images without the detriment of a significant shadow from the spectroscopic optics. This spectrometer was used to monitor the radiation damage to heme-containing P450nor crystals during X-ray irradiation in real time. The spectrometer was also used to induce the photolysis of a caged compound and subsequently monitor the formation of a substrate-bound state. The latter type of application will expand the utility of this spectrometer when combined with time-resolved- and/or cryo-trapping techniques (Bourgeois & Royant, 2005 ▸; Neutze & Moffat, 2012 ▸; Pearson & Owen, 2009 ▸; Schlichting *et al.*, 2000 ▸). One of the most powerful potential applications may be the simultaneous structural analysis and spectroscopic characterization of reaction intermediates in real time. However, it should be noted that, although the crystal angle is fixed in this study, X-ray diffraction data collection requires that the crystal be rotated, which causes spectral changes including the spectral shape and the offset shift (Owen *et al.*, 2011 ▸). Moreover, the accessible angle of crystal rotation for the spectroscopic measurement depends on the presence of the cryo-loop as well as the crystal size in terms of the light path length and cross sectional area. The white light focal size is relatively large (∅100 µm) in the present system. Therefore, it is desirable that the crystal should have a flat plane with an area of *ca* 300 µm × 300 µm, which ensures a cross sectional area of 100 µm × 100 µm within the crystal rotation range of ±60°. The problems above should always be taken into account.

## 6. Related literature   

The following references are mentioned in the supporting information: Chiu *et al.* (2006) ▸; Otwinowski & Minor (1997) ▸.

## Supplementary Material

Figs. S1 and S2, Table S1. DOI: 10.1107/S1600577515018275/rv5043sup1.pdf
