# Supplementary material for: A nearly on-axis spectroscopic system for simultaneously measuring UV–visible absorption and X-ray diffraction in the SPring-8 structural genomics beamline
Source: J Synchrotron Radiat. 2016 Jan 1;23(Pt 1):334–8. doi: 10.1107/S1600577515018275 (PMC5356500; doi:10.1107/S1600577515018275)
Supplement: Supplementary file 1 [file s-23-00334-sup1.pdf]

JOURNAL OF SYNCHROTRON RADIATION

Volume 23 (2016)

Supporting information for article:

A nearly on-axis spectroscopic system for simultaneously measuring UV-visible absorption and X-ray diffraction in the SPring-8 structural genomics beamline

Miyuki Sakaguchi, Tetsunari Kimura, Takuma Nishida, Takehiko Tosha, Hiroshi Sugimoto, Yoshihiro Yamaguchi, Sachiko Yanagisawa, Go Ueno, Hironori Murakami, Hideo Ago, Masaki Yamamoto, Takashi Ogura, Yoshitsugu Shiro and Minoru Kubo

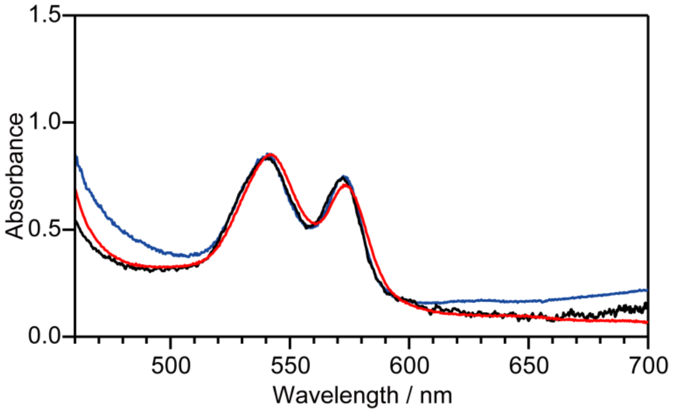

Figure S1 Visible absorption spectra of NO-bound ferric P450nor. The black line shows the spectrum of a crystal sample measured using the present spectrometer (the same spectrum as shown in Fig. 3, black), whereas the blue line the spectrum measured using the off-line microspectrometer installed previously at SPring-8 (Chiu *et al.*, 2006). The spectrum of a solution sample measured using a commercially-available spectrometer (U-3000, Hitachi) is also shown in the red line. The spectra of the crystals and the solution were measured at 100 K and room temperature, respectively. The NO-bound form was prepared using NO gas for all the samples. The linear background subtraction and intensity scaling were performed to overlay the spectra for comparison. All the spectra exhibit the two peaks, characteristic of the NO-bound form, in common, but the peak positions are slightly different between the crystal and solution samples.

Table S1 Data statistics with and without the prism shadow[1]

	– shadow	+ shadow[2]
Resolution (Å)	25.0-2.0 (25.0-10.0)	25.0-2.0 (25.0-10.0)
Completeness (%)	100 (94.8)	100 (93.7)
Redundancy	7.3 (6.2)	7.2 (4.3)

[1]The diffraction images of 180 degrees rotation collected from an orthorhombic NO-bound P450nor crystal were used for the comparison. Values in parentheses are for "the low-resolution shells". X-ray diffraction data were collected at 100 K at SPring-8/BL26B2. All the diffraction data were integrated and scaled using the HKL2000 package (Otwinowski & Minor, 1997).

[2]The diffraction spots in the blind region corresponding to the shadow area (Figure S2) were excluded from the integration using the masking tool of HKL2000.

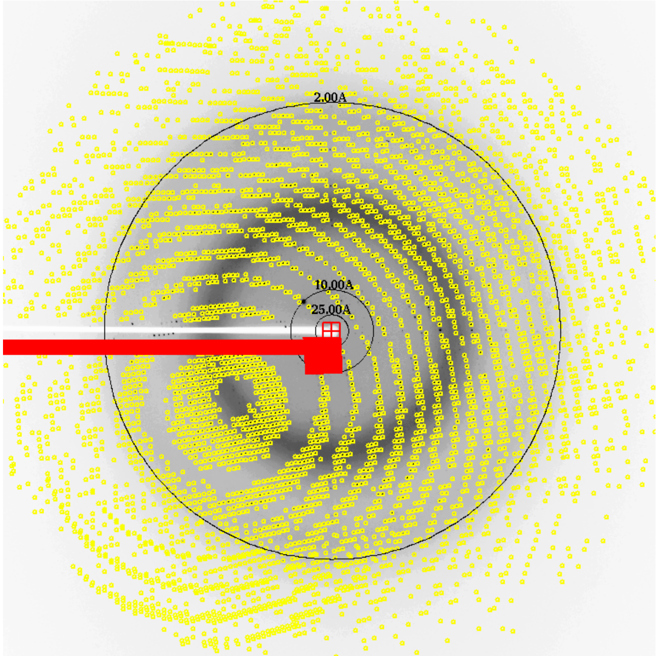

Figure S2 X-ray diffraction image of NO-bound P450nor with the blind region corresponding to the shadow area of the prism and its support rod (red). The yellow circles are the predicted reflection positions.

References

Chiu, Y.-C., Okajima, T., Murakawa, T., Uchida, M., Taki, M., Hirota, S., Kim, M., Yamaguchi, H., Kawano, Y., Kamiya, N., Kuroda, S., Hayashi, H., Yamamoto, Y. & Tanizawa, K. (2006). *Biochemistry* **45**, 4105-4120.

Otwinowski, Z. & Minor, W. (1997) *Methods Enzymol.* **276**, 307-326.
